# Supplementary material for: Performance and muscle lipogenesis of calves born to Nellore cows with different residual feed intake classification
Source: PLoS One. 2022 Jul 29;17(7):e0272236. doi: 10.1371/journal.pone.0272236 (PMC9337683; doi:10.1371/journal.pone.0272236)
Supplement: S1 Fig — (DOCX) [file pone.0272236.s001.docx]

| **Genetic value of residual feed intake (RFI) – Nellore animals born in 2014 at Instituto de Zootecnia** | | | |
| --- | --- | --- | --- |
| animal: animal identification | | |  |
| sex: 1=male; 2=female | | |  |
| year: year of birth | |  |  |
| ebv_rfi: expected breeding value for residual feed intake | | | |
|  |  |  |  |
|  |  |  |  |
| **animal** | **sex** | **year** | **ebv_rfi** |
| 2143586 | 2 | 2014 | -0,106 |
| 2143587 | 2 | 2014 | -0,080 |
| 2143588 | 2 | 2014 | -0,072 |
| 2143589 | 2 | 2014 | -0,040 |
| 2143590 | 1 | 2014 | -0,072 |
| 2143591 | 2 | 2014 | -0,163 |
| 2143592 | 2 | 2014 | -0,125 |
| 2143593 | 2 | 2014 | -0,141 |
| 2143594 | 2 | 2014 | -0,103 |
| 2143595 | 2 | 2014 | -0,036 |
| 2143596 | 1 | 2014 | -0,049 |
| 2143597 | 1 | 2014 | -0,103 |
| 2143598 | 2 | 2014 | -0,090 |
| 2143599 | 2 | 2014 | 0,041 |
| 2143600 | 1 | 2014 | -0,044 |
| 2143601 | 2 | 2014 | -0,046 |
| 2143602 | 2 | 2014 | -0,043 |
| 2143603 | 1 | 2014 | 0,048 |
| 2143604 | 1 | 2014 | -0,030 |
| 2143605 | 1 | 2014 | 0,037 |
| 2143606 | 2 | 2014 | -0,047 |
| 2143607 | 2 | 2014 | -0,058 |
| 2143608 | 1 | 2014 | -0,042 |
| 2143609 | 2 | 2014 | -0,100 |
| 2143610 | 1 | 2014 | -0,127 |
| 2143611 | 2 | 2014 | -0,074 |
| 2143612 | 1 | 2014 | 0,034 |
| 2143613 | 2 | 2014 | -0,050 |
| 2143614 | 1 | 2014 | -0,176 |
| 2143615 | 2 | 2014 | -0,011 |
| 2143616 | 2 | 2014 | 0,033 |
| 2143617 | 2 | 2014 | -0,125 |
| 2143618 | 2 | 2014 | -0,026 |
| 2143619 | 1 | 2014 | 0,114 |
| 2143620 | 2 | 2014 | -0,034 |
| 2143621 | 1 | 2014 | -0,183 |
| 2143622 | 2 | 2014 | 0,062 |
| 2143623 | 1 | 2014 | -0,081 |
| 2143624 | 2 | 2014 | -0,090 |
| 2143625 | 2 | 2014 | -0,092 |
| 2143626 | 2 | 2014 | -0,040 |
| 2143627 | 2 | 2014 | -0,063 |
| 2143628 | 1 | 2014 | -0,054 |
| 2143629 | 1 | 2014 | -0,164 |
| 2143630 | 2 | 2014 | -0,027 |
| **animal** | **sex** | **year** | **ebv_rfi** |
| 2143631 | 2 | 2014 | -0,060 |
| 2143632 | 1 | 2014 | 0,040 |
| 2143633 | 2 | 2014 | -0,056 |
| 2143634 | 2 | 2014 | -0,162 |
| 2143635 | 2 | 2014 | -0,090 |
| 2143636 | 2 | 2014 | 0,120 |
| 2143637 | 2 | 2014 | -0,017 |
| 2143638 | 1 | 2014 | 0,062 |
| 2143639 | 1 | 2014 | 0,040 |
| 2143640 | 2 | 2014 | -0,090 |
| 2143641 | 1 | 2014 | -0,112 |
| 2143642 | 2 | 2014 | 0,033 |
| 2143643 | 2 | 2014 | -0,083 |
| 2143644 | 2 | 2014 | 0,037 |
| 2143645 | 2 | 2014 | -0,011 |
| 2143646 | 2 | 2014 | -0,026 |
| 2143647 | 1 | 2014 | -0,093 |
| 2143648 | 1 | 2014 | -0,125 |
| 2143649 | 1 | 2014 | -0,090 |
| 2143650 | 2 | 2014 | -0,104 |
| 2143651 | 1 | 2014 | 0,072 |
| 2143652 | 1 | 2014 | 0,088 |
| 2143653 | 1 | 2014 | 0,001 |
| 2143654 | 1 | 2014 | -0,046 |
| 2143655 | 2 | 2014 | -0,080 |
| 2143656 | 1 | 2014 | -0,074 |
| 2143657 | 1 | 2014 | -0,075 |
| 2143658 | 1 | 2014 | -0,036 |
| 2143659 | 2 | 2014 | -0,134 |
| 2143660 | 1 | 2014 | -0,004 |
| 2143661 | 1 | 2014 | -0,055 |
| 2143662 | 2 | 2014 | -0,088 |
| 2143663 | 2 | 2014 | -0,098 |
| 2143664 | 1 | 2014 | -0,180 |
| 2143665 | 1 | 2014 | -0,128 |
| 2143666 | 1 | 2014 | 0,119 |
| 2143667 | 1 | 2014 | -0,151 |
| 2143668 | 1 | 2014 | -0,099 |
| 2143669 | 2 | 2014 | -0,066 |
| 2143670 | 1 | 2014 | 0,006 |
| 2143671 | 1 | 2014 | -0,100 |
| 2143672 | 1 | 2014 | -0,044 |
| 2143673 | 1 | 2014 | -0,049 |
| 2143674 | 2 | 2014 | -0,108 |
| 2143675 | 1 | 2014 | -0,051 |
| 2143676 | 1 | 2014 | 0,013 |
| 2143677 | 2 | 2014 | 0,026 |
| 2143678 | 2 | 2014 | -0,067 |
| 2143679 | 1 | 2014 | -0,107 |
| 2143680 | 2 | 2014 | 0,011 |
| 2143681 | 1 | 2014 | 0,098 |
| 2143682 | 1 | 2014 | -0,081 |
| 2143683 | 2 | 2014 | -0,035 |
| **animal** | **sex** | **year** | **ebv_rfi** |
| 2143684 | 1 | 2014 | -0,063 |
| 2143685 | 1 | 2014 | 0,036 |
| 2143686 | 2 | 2014 | -0,070 |
| 2143687 | 2 | 2014 | -0,072 |
| 2143688 | 1 | 2014 | -0,089 |
| 2143689 | 2 | 2014 | -0,133 |
| 2143690 | 1 | 2014 | 0,074 |
| 2143691 | 2 | 2014 | -0,031 |
| 2143692 | 2 | 2014 | -0,083 |
| 2143693 | 2 | 2014 | -0,088 |
| 2143694 | 1 | 2014 | -0,094 |
| 2143695 | 2 | 2014 | -0,141 |
| 2143696 | 1 | 2014 | 0,023 |
| 2143697 | 2 | 2014 | -0,114 |
| 2143698 | 1 | 2014 | 0,144 |
| 2143699 | 2 | 2014 | 0,030 |
| 2143700 | 2 | 2014 | -0,062 |
| 2143701 | 2 | 2014 | -0,031 |
| 2143702 | 2 | 2014 | -0,021 |
| 2143703 | 1 | 2014 | 0,039 |
| 2143704 | 2 | 2014 | 0,210 |
| 2143705 | 1 | 2014 | 0,039 |
| 2143706 | 1 | 2014 | 0,062 |
| 2143707 | 2 | 2014 | -0,014 |
| 2143708 | 2 | 2014 | 0,085 |
| 2143709 | 2 | 2014 | -0,151 |
| 2143710 | 1 | 2014 | -0,074 |
| 2143711 | 2 | 2014 | -0,183 |
| 2143712 | 1 | 2014 | 0,067 |
| 2143713 | 1 | 2014 | -0,065 |
| 2143714 | 2 | 2014 | -0,139 |
| 2143715 | 2 | 2014 | -0,095 |
| 2143716 | 1 | 2014 | -0,137 |
| 2143717 | 2 | 2014 | -0,085 |
| 2143718 | 1 | 2014 | -0,064 |
| 2143719 | 1 | 2014 | -0,029 |
| 2143720 | 2 | 2014 | -0,140 |
| 2143721 | 1 | 2014 | 0,057 |
| 2143722 | 2 | 2014 | -0,018 |
| 2143723 | 1 | 2014 | -0,081 |
| 2143724 | 1 | 2014 | -0,056 |
| 2143725 | 2 | 2014 | -0,030 |
| 2143726 | 2 | 2014 | -0,044 |
| 2143727 | 2 | 2014 | -0,026 |
| 2143728 | 2 | 2014 | 0,025 |
| 2143729 | 2 | 2014 | 0,001 |
| 2143730 | 1 | 2014 | -0,142 |
| 2143731 | 2 | 2014 | -0,099 |
| 2143732 | 1 | 2014 | -0,138 |
| 2143733 | 2 | 2014 | -0,020 |
| 2143734 | 2 | 2014 | -0,010 |
| 2143735 | 2 | 2014 | -0,013 |
| 2143736 | 2 | 2014 | -0,021 |
| **animal** | **sex** | **year** | **ebv_rfi** |
| 2143737 | 2 | 2014 | -0,089 |
| 2143738 | 2 | 2014 | -0,017 |
| 2143739 | 1 | 2014 | -0,030 |
| 2143740 | 2 | 2014 | -0,063 |
| 2143741 | 2 | 2014 | -0,062 |
| 2143742 | 1 | 2014 | -0,122 |
| 2143743 | 1 | 2014 | -0,150 |
| 2143744 | 2 | 2014 | -0,101 |
| 2143745 | 1 | 2014 | -0,030 |
| 2143746 | 1 | 2014 | -0,135 |
| 2143747 | 1 | 2014 | -0,045 |
| 2143748 | 1 | 2014 | 0,037 |
| 2143749 | 1 | 2014 | -0,047 |
| 2143750 | 1 | 2014 | -0,019 |
| 2143751 | 2 | 2014 | 0,043 |
| 2143752 | 2 | 2014 | -0,036 |
| 2143753 | 1 | 2014 | -0,157 |
| 2143754 | 2 | 2014 | -0,155 |
| 2143755 | 2 | 2014 | -0,161 |
| 2143756 | 2 | 2014 | -0,033 |
| 2143757 | 2 | 2014 | -0,134 |
| 2143758 | 1 | 2014 | -0,181 |
| 2143759 | 1 | 2014 | 0,022 |
| 2143760 | 1 | 2014 | 0,042 |
| 2143761 | 1 | 2014 | 0,028 |
| 2143762 | 1 | 2014 | -0,177 |
| 2143763 | 2 | 2014 | -0,032 |
| 2143764 | 2 | 2014 | -0,083 |
| 2143765 | 1 | 2014 | -0,066 |
| 2143766 | 2 | 2014 | -0,146 |
| 2143767 | 1 | 2014 | -0,032 |
| 2143768 | 2 | 2014 | 0,035 |
| 2143769 | 1 | 2014 | -0,059 |
| 2143770 | 2 | 2014 | -0,077 |
| 2143771 | 1 | 2014 | -0,157 |
| 2143772 | 2 | 2014 | 0,169 |
| 2143773 | 1 | 2014 | -0,122 |
| 2143774 | 1 | 2014 | -0,050 |
| 2143775 | 2 | 2014 | 0,038 |
| 2143776 | 1 | 2014 | -0,057 |
| 2143777 | 2 | 2014 | -0,051 |
| 2143778 | 2 | 2014 | -0,082 |
| 2143779 | 1 | 2014 | -0,110 |
| 2143780 | 1 | 2014 | 0,067 |
| 2143781 | 1 | 2014 | -0,174 |
| 2143782 | 2 | 2014 | 0,085 |
| 2143783 | 2 | 2014 | 0,058 |
| 2143784 | 1 | 2014 | -0,087 |
| 2143785 | 1 | 2014 | -0,271 |
| 2143786 | 2 | 2014 | 0,031 |
| 2143787 | 1 | 2014 | -0,081 |
| 2143788 | 2 | 2014 | 0,035 |
| 2143789 | 1 | 2014 | -0,125 |
| **animal** | **sex** | **year** | **ebv_rfi** |
| 2143790 | 1 | 2014 | -0,071 |
| 2143791 | 1 | 2014 | -0,013 |
| 2143792 | 2 | 2014 | -0,195 |
| 2143793 | 2 | 2014 | 0,013 |
| 2143794 | 2 | 2014 | 0,051 |
| 2143795 | 2 | 2014 | 0,028 |
| 2143796 | 1 | 2014 | -0,120 |
| 2143797 | 1 | 2014 | 0,121 |
| 2143798 | 2 | 2014 | 0,017 |
| 2143799 | 1 | 2014 | -0,048 |
| 2143800 | 1 | 2014 | -0,200 |
| 2143801 | 1 | 2014 | -0,098 |
| 2143802 | 2 | 2014 | -0,162 |
| 2143803 | 2 | 2014 | 0,067 |
| 2143804 | 1 | 2014 | -0,091 |
| 2143805 | 1 | 2014 | 0,110 |
| 2143806 | 2 | 2014 | 0,134 |
| 2143807 | 2 | 2014 | 0,002 |
| 2143808 | 2 | 2014 | -0,019 |
| 2143809 | 1 | 2014 | -0,139 |
| 2143810 | 2 | 2014 | -0,198 |
| 2143811 | 2 | 2014 | -0,050 |
| 2143812 | 2 | 2014 | -0,052 |
| 2143813 | 2 | 2014 | -0,107 |
| 2143814 | 1 | 2014 | 0,038 |
| 2143815 | 2 | 2014 | 0,051 |
| 2143816 | 2 | 2014 | -0,114 |
| 2143817 | 1 | 2014 | 0,009 |
| 2143818 | 2 | 2014 | -0,102 |
| 2143819 | 1 | 2014 | -0,036 |
| 2143820 | 2 | 2014 | -0,101 |
| 2143821 | 1 | 2014 | 0,008 |
| 2143822 | 1 | 2014 | 0,004 |
| 2143823 | 1 | 2014 | 0,039 |
| 2143824 | 1 | 2014 | 0,121 |
| 2143825 | 2 | 2014 | 0,006 |
| 2143826 | 2 | 2014 | -0,164 |
| 2143827 | 1 | 2014 | -0,119 |
| 2143828 | 2 | 2014 | -0,022 |
| 2143829 | 1 | 2014 | -0,049 |
| 2143830 | 2 | 2014 | -0,060 |
| 2143831 | 1 | 2014 | -0,050 |
| 2143832 | 2 | 2014 | -0,020 |
| 2143833 | 2 | 2014 | -0,030 |
| 2143834 | 1 | 2014 | -0,064 |
| 2143835 | 1 | 2014 | -0,128 |
| 2143836 | 2 | 2014 | -0,080 |
| 2143837 | 1 | 2014 | -0,203 |
| 2143838 | 2 | 2014 | -0,044 |
| 2143839 | 2 | 2014 | -0,122 |
| 2143840 | 2 | 2014 | -0,164 |
| 2143841 | 1 | 2014 | -0,191 |
| 2143842 | 1 | 2014 | -0,048 |
| **animal** | **sex** | **year** | **ebv_rfi** |
| 2143843 | 2 | 2014 | 0,009 |
| 2143844 | 2 | 2014 | 0,083 |
| 2143845 | 2 | 2014 | -0,089 |
| 2143846 | 2 | 2014 | -0,126 |
| 2143847 | 1 | 2014 | -0,169 |
| 2143848 | 1 | 2014 | -0,135 |
| 2143849 | 1 | 2014 | -0,150 |
| 2143850 | 2 | 2014 | 0,011 |
| 2143851 | 2 | 2014 | -0,193 |
| 2143852 | 2 | 2014 | -0,102 |
| 2143853 | 1 | 2014 | -0,052 |
| 2143854 | 2 | 2014 | -0,065 |
| 2143855 | 1 | 2014 | -0,156 |
| 2143856 | 2 | 2014 | -0,082 |
| 2143857 | 2 | 2014 | 0,064 |
| 2143858 | 1 | 2014 | 0,077 |
| 2143859 | 2 | 2014 | -0,015 |
| 2143860 | 2 | 2014 | 0,009 |
| 2143861 | 1 | 2014 | -0,217 |
| 2143862 | 2 | 2014 | -0,113 |
| 2143863 | 1 | 2014 | -0,113 |
| 2143864 | 1 | 2014 | 0,082 |
| 2143865 | 2 | 2014 | -0,124 |
| 2143866 | 2 | 2014 | -0,095 |
| 2143867 | 2 | 2014 | -0,059 |
| 2143868 | 2 | 2014 | -0,087 |
| 2143869 | 1 | 2014 | -0,018 |
| 2143870 | 1 | 2014 | -0,138 |
| 2143871 | 2 | 2014 | 0,081 |
| 2143872 | 2 | 2014 | 0,017 |
| 2143873 | 1 | 2014 | -0,169 |
| 2143874 | 1 | 2014 | -0,123 |
| 2143875 | 2 | 2014 | -0,020 |
| 2143876 | 2 | 2014 | 0,118 |
| 2143877 | 2 | 2014 | -0,100 |
| 2143878 | 2 | 2014 | -0,082 |
| 2143879 | 1 | 2014 | -0,092 |
| 2143880 | 1 | 2014 | 0,015 |
| 2143881 | 2 | 2014 | 0,021 |
| 2143882 | 2 | 2014 | 0,077 |
| 2143883 | 2 | 2014 | -0,043 |
| 2143884 | 1 | 2014 | -0,119 |
| 2143885 | 1 | 2014 | -0,102 |
| 2143886 | 2 | 2014 | 0,024 |
| 2143887 | 2 | 2014 | -0,115 |
| 2143888 | 1 | 2014 | 0,092 |
| 2143889 | 2 | 2014 | -0,140 |
| 2143890 | 1 | 2014 | -0,130 |
| 2143891 | 1 | 2014 | -0,297 |
| 2143892 | 2 | 2014 | -0,139 |
| 2143893 | 1 | 2014 | -0,151 |
| 2143894 | 2 | 2014 | -0,061 |
| 2143895 | 1 | 2014 | 0,008 |
| **animal** | **sex** | **year** | **ebv_rfi** |
| 2143896 | 2 | 2014 | -0,038 |
| 2143897 | 2 | 2014 | -0,031 |
| 2143898 | 1 | 2014 | -0,071 |
| 2143899 | 1 | 2014 | 0,077 |
| 2143900 | 1 | 2014 | 0,009 |
