# Supplementary material for: Performance and muscle lipogenesis of calves born to Nellore cows with different residual feed intake classification
Source: PLoS One. 2022 Jul 29;17(7):e0272236. doi: 10.1371/journal.pone.0272236 (PMC9337683; doi:10.1371/journal.pone.0272236)
Supplement: S2 Fig — (DOCX) [file pone.0272236.s002.docx]

| **Cow-calf pair performance data** | | | | |  |  |  |  |  |  |  |
| --- | --- | --- | --- | --- | --- | --- | --- | --- | --- | --- | --- |
| calf: calf identification | | |  |  |  |  |  |  |  |  |  |
| sex: 1=male; 2=female | | |  |  |  |  |  |  |  |  |  |
| day: calf day of birth | | |  |  |  |  |  |  |  |  |  |
| month: calf month of birth | | | |  |  |  |  |  |  |  |  |
| year: calf year of birth | | |  |  |  |  |  |  |  |  |  |
| cow: cow identification | | |  |  |  |  |  |  |  |  |  |
| rfi class: 1=negative RFI; 2=positive RFI | | | | |  |  |  |  |  |  |  |
| c_bw1: cow body weight at the first month of the second trimester of gestation | | | | | | | | |  |  |  |
| c_bw2: cow body weight at the second month of the second trimester of gestation | | | | | | | | | |  |  |
| c_bw3: cow body weight at the third month of the second trimester of gestation | | | | | | | | |  |  |  |
| c_wbw: cow weaning body weight | | | | |  |  |  |  |  |  |  |
| c_adg: cow average daily weight gain | | | | |  |  |  |  |  |  |  |
| c_ft: cow rib fat thickness | | | |  |  |  |  |  |  |  |  |
| c_rea: cow rib eye area | | |  |  |  |  |  |  |  |  |  |
| c_bft: cow back fat thickness | | | |  |  |  |  |  |  |  |  |
| ca_bbw: calf birth body weight | | | |  |  |  |  |  |  |  |  |
| ca_bw1: calf body weight at 120 days of age | | | | | |  |  |  |  |  |  |
| ca_bw2: calf body weight at 210 days of age | | | | | |  |  |  |  |  |  |
| ca_wbw: calf weaning body weight | | | | |  |  |  |  |  |  |  |
| ca_adg: calf average daily weight gain | | | | |  |  |  |  |  |  |  |
| ca_ft: calf rib fat thickness | | | |  |  |  |  |  |  |  |  |
| ca_rea: calf rib eye area | | | |  |  |  |  |  |  |  |  |
| ca_bft: calf back fat thickness | | | |  |  |  |  |  |  |  |  |
|  |  |  |  |  |  |  |  |  |  |  |  |
| **calf** | **sex** | **day** | **month** | **year** | **cow** | **rfi class** | **c_bw1** | **c_bw2** | **c_bw3** | **c_wbw** | **c_adg** |
| 2174679 | 2 | 27 | 9 | 2017 | 2143587 | 1 | 517 | 547 | 564 | 648 | 0,719 |
| 2174671 | 2 | 25 | 9 | 2017 | 2143588 | 1 | 565 | 587 | 602 | 680 | 0,565 |
| 2174678 | 2 | 27 | 9 | 2017 | 2143598 | 2 | 546 | 555 | 557 | 629 | 0,165 |
| 2174715 | 1 | 3 | 10 | 2017 | 2143599 | 2 | 528 | 506 | 560 | 635 | 0,501 |
| 2174670 | 1 | 25 | 9 | 2017 | 2143606 | 2 | 536 | 563 | 571 | 656 | 0,426 |
| 2171824 | 1 | 8 | 11 | 2017 | 2143607 | 2 | 431 | 444 | 463 | 543 | 0,496 |
| 2174620 | 1 | 18 | 9 | 2017 | 2143637 | 2 | 444 | 453 | 459 | 548 | 0,239 |
| 2174835 | 2 | 12 | 11 | 2017 | 2143678 | 2 | 490 | 515 | 522 | 740 | 0,381 |
| 2174714 | 1 | 3 | 10 | 2017 | 2143709 | 1 | 504 | 531 | 532 | 641 | 0,418 |
| 2174709 | 1 | 2 | 10 | 2017 | 2143711 | 1 | 490 | 516 | 518 | 638 | 0,438 |
| 2174853 | 1 | 19 | 11 | 2017 | 2143715 | 2 | 458 | 470 | 475 | 577 | 0,202 |
| 2174852 | 2 | 19 | 11 | 2017 | 2143735 | 2 | 586 | 587 | 595 | 626 | 0,098 |
| 2174703 | 2 | 29 | 9 | 2017 | 2143738 | 2 | 581 | 608 | 621 | 715 | 0,620 |
| 2171826 | 2 | 19 | 11 | 2017 | 2143740 | 2 | 473 | 492 | 498 | 568 | 0,306 |
| 2174635 | 2 | 21 | 9 | 2017 | 2143741 | 2 | 529 | 553 | 574 | 644 | 0,685 |
| 2174637 | 1 | 21 | 9 | 2017 | 2143754 | 1 | 525 | 539 | 548 | 664 | 0,351 |
| 2174820 | 2 | 4 | 11 | 2017 | 2143755 | 1 | 412 | 422 | 422 | 535 | 0,117 |
| 2174836 | 2 | 12 | 11 | 2017 | 2143766 | 1 | 517 | 533 | 545 | 618 | 0,340 |
| 2174668 | 2 | 25 | 9 | 2017 | 2143778 | 2 | 421 | 436 | 444 | 492 | 0,340 |
| 2174636 | 2 | 21 | 9 | 2017 | 2143802 | 1 | 513 | 521 | 523 | 579 | 0,160 |
| 2174669 | 1 | 25 | 9 | 2017 | 2143818 | 1 | 525 | 545 | 567 | 622 | 0,648 |
| 2174827 | 2 | 9 | 11 | 2017 | 2143838 | 2 | 567 | 571 | 581 | 621 | 0,169 |
| 2171825 | 1 | 15 | 11 | 2017 | 2143877 | 2 | 448 | 457 | 473 | 609 | 0,306 |
| 2174723 | 1 | 4 | 10 | 2017 | 2143889 | 1 | 454 | 471 | 481 | 622 | 0,421 |
| 2174834 | 1 | 12 | 11 | 2017 | 2143894 | 1 | 458 | 479 | 492 | 535 | 0,415 |
| 2184905 | 1 | 3 | 9 | 2018 | 2143586 | 2 | 637 | 652 | 663 | 514 | 0,413 |
| 2185041 | 1 | 13 | 10 | 2018 | 2143588 | 1 | 639 | 676 | 705 | 553 | 1,028 |
| 2184898 | 1 | 30 | 8 | 2018 | 2143598 | 2 | 592 | 621 | 631 | 569 | 0,613 |
| 2181832 | 1 | 27 | 8 | 2018 | 2143611 | 2 | 598 | 607 | 620 | 520 | 0,335 |
| 2184894 | 2 | 27 | 8 | 2018 | 2143627 | 1 | 711 | 727 | 739 | 591 | 0,442 |
| 2184890 | 1 | 21 | 8 | 2018 | 2143637 | 2 | 515 | 541 | 558 | 503 | 0,669 |
| 2181831 | 2 | 27 | 8 | 2018 | 2143687 | 2 | 616 | 627 | 641 | . | 0,388 |
| **calf** | **sex** | **day** | **month** | **year** | **cow** | **rfi class** | **c_bw1** | **c_bw2** | **c_bw3** | **c_wbw** | **c_adg** |
| 2184901 | 1 | 30 | 8 | 2018 | 2143709 | 1 | 612 | 634 | 665 | . | 0,828 |
| 2184899 | 1 | 30 | 8 | 2018 | 2143711 | 1 | 606 | 631 | 629 | 543 | 0,360 |
| 2185011 | 1 | 4 | 10 | 2018 | 2143715 | 2 | 547 | 558 | 566 | 476 | 0,293 |
| 2184892 | 2 | 27 | 8 | 2018 | 2143720 | 1 | 609 | 621 | 629 | 542 | 0,317 |
| 2184893 | 1 | 27 | 8 | 2018 | 2143729 | 2 | 550 | 558 | 562 | 471 | 0,192 |
| 2184906 | 1 | 3 | 9 | 2018 | 2143734 | 2 | 594 | 607 | 619 | 528 | 0,400 |
| 2184889 | 2 | 20 | 8 | 2018 | 2143741 | 2 | 607 | 638 | 660 | 522 | 0,820 |
| 2184903 | 2 | 3 | 9 | 2018 | 2143754 | 1 | 620 | 658 | 684 | 586 | 1,001 |
| 2185040 | 1 | 13 | 10 | 2018 | 2143755 | 1 | 476 | 498 | 527 | 472 | 0,800 |
| 2184887 | 2 | 16 | 8 | 2018 | 2143756 | 2 | 576 | 586 | 596 | 486 | 0,320 |
| 2184888 | 1 | 16 | 8 | 2018 | 2143756 | 2 | 576 | 586 | 597 | 486 | 0,327 |
| 2184896 | 1 | 28 | 8 | 2018 | 2143763 | 2 | 639 | 645 | 648 | . | 0,147 |
| 2184904 | 2 | 3 | 9 | 2018 | 2143793 | 2 | 590 | 597 | 604 | 513 | 0,222 |
| 2184900 | 1 | 30 | 8 | 2018 | 2143802 | 1 | 566 | 571 | 603 | 486 | 0,581 |
| 2184895 | 1 | 27 | 8 | 2018 | 2143811 | 2 | 635 | 640 | 656 | 540 | 0,336 |
| 2184891 | 1 | 27 | 8 | 2018 | 2143836 | 1 | 663 | 676 | 689 | 563 | 0,410 |
| 2184910 | 1 | 7 | 9 | 2018 | 2143845 | 2 | 579 | 606 | 610 | 591 | 0,497 |
| 2184897 | 2 | 30 | 8 | 2018 | 2143862 | 1 | 561 | 575 | 595 | 488 | 0,542 |
| 2181837 | 1 | 10 | 10 | 2018 | 2143877 | 2 | 544 | 579 | 598 | 484 | 0,836 |
| 2181836 | 1 | 6 | 10 | 2018 | 2143892 | 1 | 576 | 594 | 612 | 514 | 0,562 |
| 2181830 | 2 | 27 | 8 | 2018 | 2143897 | 1 | 586 | 597 | 608 | 539 | 0,348 |

| **c_ft** | **c_rea** | **c_bft** | **ca_bbw** | **ca_bw1** | **ca_bw2** | **ca_wbw** | **ca_wa** | **ca_adg** |
| --- | --- | --- | --- | --- | --- | --- | --- | --- |
| 6,3 | 76,8 | 5,9 | 33 | 148 | 258 | 275 | 231 | 1,072 |
| 4,1 | 78,0 | 6,6 | 28 | 164 | 277 | 304 | 233 | 1,185 |
| 8,9 | 87,7 | 11,8 | 33 | 161 | 263 | 290 | 231 | 1,093 |
| 8,8 | 82,1 | 9,9 | 34 | 162 | 264 | 282 | 225 | 1,096 |
| 6,2 | 74,5 | 7,8 | 33 | 147 | 244 | 291 | 233 | 1,004 |
| 5,9 | 78,9 | 7,6 | 35 | 154 | 248 | 231 | 189 | 1,016 |
| 4,3 | 75,1 | 4,7 | 34 | 150 | 253 | 282 | 240 | 1,045 |
| 4,6 | 64,3 | 5,4 | 30 | . | . | . | . | . |
| 4,2 | 76,3 | 5,9 | 36 | 151 | 239 | 256 | 225 | 0,965 |
| 4,2 | 83,7 | 5,5 | 45 | 175 | 287 | 314 | 226 | 1,152 |
| 6,2 | 87,6 | 7,4 | 34 | 150 | 214 | 196 | 178 | 0,858 |
| 5,1 | 83,3 | 7,6 | 31 | 144 | 231 | 202 | 178 | 0,951 |
| 7,8 | 81,4 | 9,7 | 30 | 116 | 209 | 240 | 229 | 0,855 |
| 8,1 | 84,8 | 9,1 | 37 | 165 | 256 | 223 | 178 | 1,041 |
| 6,2 | 75,3 | 8,6 | 29 | 144 | 237 | 270 | 237 | 0,992 |
| 4,1 | 74,4 | 6,6 | 38 | 153 | 258 | 291 | 237 | 1,045 |
| 4,0 | 78,7 | 5,3 | 34 | 147 | 232 | 214 | 193 | 0,944 |
| 7,2 | 81,3 | 8,5 | 32 | 152 | 232 | 222 | 185 | 0,953 |
| 2,8 | 77,2 | 3,1 | 26 | 130 | 217 | 246 | 233 | 0,912 |
| 6,0 | 74,4 | 8,0 | 35 | 158 | 259 | 288 | 237 | 1,066 |
| 5,9 | 76,2 | 7,8 | 31 | 175 | 289 | 314 | 233 | 1,227 |
| 8,4 | 85,3 | 12,3 | 26 | 122 | 188 | 177 | 188 | 0,772 |
| 5,0 | 82,0 | 6,6 | 37 | 182 | 283 | 253 | 182 | 1,171 |
| 4,1 | 83,6 | 5,0 | 35 | 165 | 272 | 275 | 224 | 1,127 |
| 4,1 | 82,2 | 6,2 | 44 | 200 | 308 | 283 | 185 | 1,258 |
| 8,0 | 86,7 | 9,5 | 37 | 133 | 200 | 230 | 249 | 0,808 |
| 5,7 | 82,3 | 9,1 | 39 | 139 | 219 | 216 | 209 | 1,065 |
| 10,2 | 86,8 | 15,8 | 43 | 98 | . | . | . | . |
| 8,6 | 82,7 | 11,4 | 32 | 118 | 188 | 220 | 256 | 0,743 |
| 6,7 | 74,4 | 8,4 | 28 | 101 | 157 | 184 | 256 | 0,622 |
| 5,8 | 80,7 | 7,9 | 35 | 120 | 195 | 232 | 262 | 0,752 |
| 7,1 | 93,3 | 12,2 | 30 | 106 | 137 | 159 | 256 | 0,542 |
| 7,4 | 90,3 | 11,2 | 39 | 113 | 143 | 163 | 253 | 0,573 |
| 6,8 | 81,6 | 9,7 | 39 | 138 | 217 | 251 | 253 | 0,868 |
| 8,0 | 87,4 | 10,0 | 30 | 119 | 185 | 189 | 218 | 0,862 |
| **c_ft** | **c_rea** | **c_bft** | **ca_bbw** | **ca_bw1** | **ca_bw2** | **ca_wbw** | **ca_wa** | **ca_adg** |
| 11,4 | 80,3 | 10,2 | 28 | 111 | 168 | 197 | 256 | 0,665 |
| 7,5 | 75,2 | 10,3 | 28 | 117 | 187 | 220 | 256 | 0,741 |
| 8,9 | 83,6 | 10,0 | 46 | 128 | 179 | 201 | 249 | 0,735 |
| 11,0 | 89,6 | 12,7 | 32 | 121 | 199 | 239 | 263 | 0,766 |
| 8,1 | 82,9 | 11,6 | 34 | 108 | 154 | 175 | 249 | 0,628 |
| 8,7 | 82,7 | 8,0 | 35 | 120 | 200 | 197 | 209 | 0,972 |
| 8,4 | 76,1 | 10,4 | 22 | 100 | 160 | 195 | 267 | 0,605 |
| 8,4 | 76,1 | 10,4 | 20 | 85 | 142 | 173 | 267 | 0,537 |
| 9,0 | 75,0 | 9,1 | 37 | 116 | 194 | 225 | 255 | 0,769 |
| 9,4 | 76,3 | 10,9 | 38 | 144 | 223 | 255 | 249 | 0,907 |
| 9,5 | 84,0 | 11,4 | 43 | 140 | 219 | 253 | 253 | 0,878 |
| 7,0 | 75,0 | 8,4 | 27 | 130 | 202 | 238 | 256 | 0,799 |
| 8,5 | 82,3 | 9,0 | 28 | 104 | 177 | 207 | 256 | 0,698 |
| 12,2 | 83,2 | 11,0 | 40 | 125 | 193 | 216 | 245 | 0,796 |
| 10,4 | 81,7 | 11,5 | 31 | 122 | 175 | 202 | 253 | 0,699 |
| 9,2 | 78,4 | 10,5 | 32 | 104 | 188 | 187 | 212 | 0,898 |
| 7,2 | 73,4 | 10,4 | 37 | 117 | 183 | 184 | 216 | 0,869 |
| 9,2 | 82,0 | 10,6 | 28 | 116 | 191 | 224 | 256 | 0,754 |

| **ca_ft** | **ca_rea** | **ca_bft** | **ca_pe** |
| --- | --- | --- | --- |
| 2,9 | 48,3 | 8,6 | 37,1 |
| 3,5 | 46,7 | 8,3 | 38,8 |
| 3,0 | 47,5 | 7,5 | 40,3 |
| 2,9 | 49,8 | 5,3 | 40,0 |
| 2,0 | 46,2 | 4,9 | 38,4 |
| 2,3 | 39,4 | 4,9 | 46,7 |
| 2,0 | 51,0 | 5,3 | 43,1 |
| . | . | . | . |
| 1,6 | 40,7 | 5,3 | 35,9 |
| 1,5 | 50,7 | 5,3 | 44,0 |
| 0,0 | 39,2 | 3,4 | 40,0 |
| 2,0 | 40,3 | 4,5 | 37,9 |
| 1,6 | 41,1 | 4,9 | 29,6 |
| 1,3 | 38,0 | 6,0 | 46,2 |
| 3,6 | 55,7 | 7,5 | 35,7 |
| 2,3 | 43,8 | 5,6 | 37,1 |
| 2,0 | 40,4 | 4,9 | 42,9 |
| 3,8 | 43,0 | 8,3 | 40,4 |
| 1,3 | 50,5 | 4,5 | 43,4 |
| 2,7 | 45,9 | 6,4 | 42,2 |
| 2,9 | 59,1 | 6,4 | 43,8 |
| 2,0 | 38,2 | 4,1 | 31,5 |
| 1,3 | 50,0 | 3,8 | 47,6 |
| 2,9 | 56,7 | 6,0 | 40,0 |
| 2,9 | 40,7 | 5,6 | 59,5 |
| 1,9 | 42,7 | 3,0 | 35,6 |
| 0,8 | 44,6 | 2,3 | 38,2 |
| . | . | . | . |
| 1,1 | 37,1 | 2,3 | 32,7 |
| 1,5 | 21,3 | 2,6 | 24,0 |
| 1,9 | 38,3 | 2,6 | 34,6 |
| 0,8 | 25,5 | 2,6 | . |
| 0,0 | 24,3 | 1,1 | . |
| 0,8 | 40,0 | 2,3 | 36,1 |
| 1,5 | 30,1 | 2,6 | 37,0 |
| 1,9 | 41,4 | 1,9 | 28,1 |
| **ca_ft** | **ca_rea** | **ca_bft** | **ca_pe** |
| 2,3 | 30,8 | 2,6 | 36,2 |
| 0,8 | 36,2 | 1,5 | 29,8 |
| 1,1 | 38,7 | 3,8 | 34,3 |
| 0,8 | 27,9 | 1,5 | 23,6 |
| 0,8 | 34,3 | 2,3 | 40,8 |
| 1,1 | 30,6 | 2,3 | 29,8 |
| 1,9 | 29,1 | 3,0 | 26,4 |
| 1,1 | 35,8 | 2,3 | . |
| 2,3 | 42,4 | 3,8 | 39,6 |
| 1,1 | 45,3 | 1,5 | 40,5 |
| 1,5 | 41,5 | 2,3 | 34,3 |
| 1,5 | 38,5 | 2,3 | 28,5 |
| 1,1 | 31,0 | 3,0 | 29,5 |
| 1,5 | 33,7 | 2,6 | 32,3 |
| 0,4 | 32,2 | 2,3 | 37,1 |
| 1,5 | 27,5 | 1,5 | 33,6 |
| 1,1 | 38,3 | 2,6 | 32,2 |
