# Supplementary material for: Performance and muscle lipogenesis of calves born to Nellore cows with different residual feed intake classification
Source: PLoS One. 2022 Jul 29;17(7):e0272236. doi: 10.1371/journal.pone.0272236 (PMC9337683; doi:10.1371/journal.pone.0272236)
Supplement: Attachment — Submitted filename: Paz et al 2022_responses to reviewers.docx [file pone.0272236.s003.docx]

**RESPONSES TO REVIEWERS**

Manuscript ID PONE-D-21-37544:

"Performance and muscle lipogenesis of calves born to Nellore cows with different residual feed intake classification"

Dear Dr. Marcio Duarte,

We appreciated all the efforts you and the reviewers made to improve the quality of this manuscript. Below are the responses for each comment and suggestion made. The changes in the text are highlighted using Word track changes tool in marked-up copy.

Thanks a lot,

The authors

Reviewer(s)' Comments to Author:

**Reviewer: 1**

**Comments to the Author**

Overall considerations: This study evaluates the effects of maternal RFI classification on calf gene expression and growth performance. This reviewer believes authors should pay close attention to the statistical analyses, description of methods, and writing style. The manuscript is lacking flow. For example, introduction does not address well the research project and the abstract does not reflect the studies performed. It does not mention that 2 separately studies were conducted. Regarding the statistics, for instance, AI is included in the fixed model when it was not supposed to be since researchers were not testing different protocols. In fact, the AI protocol was the same for every cow, including the sire used. Therefore, no needed to be included in the fixed model. This reviewer thinks that both experiments should be analyzed as a single project, generating the interaction between feeding system x RFI. For the calf data, sex was included in the fixed term, why an interaction was not tested then (maternal RFI|SEX)? P-values for sex or AI were not even reported even though they were in the fixed model. The gene expression data could have been analyzed as a repeated measure; therefore, conclusion could be drawn when comparing the pre-weaning vs. post-weaning values. The introduction section can be improved by stating why testing different feeding systems are needed. It would justify the 2 experiments. Materials and methods are missing important information about animals/pen, days of the experiment and so on. Table 5 is not needed and could be removed from the manuscript. English/written style could be improved. This reviewer suggests authors to improve the writing style, table descriptions, and statistics analyses. Some other detailed information can be found below.

**RESPONSE:** We appreciated all the efforts you made to improve the quality of this manuscript. Thank you. As suggested, we made a better description of statistical analyzes, methodology and writing style to improve the understanding of the manuscript. A previous approach of the data was carried out having both years analyzed as a single experiment, however the present approach was more efficient in showing the effect of the cow RFI class on the lipogenesis of calves, which is the main objective of the present study. The description of the two studies is present in the abstract, however, due to the limitation of 300 words in this section, there is no space for it to be further explored. AI was included as fixed effect in the model because we had two AI protocols in each year (in the same breeding season), which implies a difference of approximately 40 days of age between calves from the first and second protocols. Calf sex was included as fixed effect in the model because there is a considerable difference in body weight between males and females at the same age. The AI protocol and sex effects inclusion in the model had the objective of adjustment for these differences, not the objective of comparing AI protocols or calves’ sex, for this reason P-values were not presented. The maternal RFI x calf sex and other interactions were tested, and because they were not significant, were excluded from the model. This information is present in the statistical analysis section. Gene expression data was not analyzed as repeated measures design, because it is important to separate the fetal/neonatal and pre-weaning periods in the expression of genes related to lipid metabolism. We do not have data regarding post-weaning period. The collections ended when the calves were weaned. Overall, we improved writing style and included the missing information as suggested.

Title: This reviewer suggests changing the title to: “Performance and muscle lipogenesis of calves born to Nellore cows with different residual feed intake classification”.

**RESPONSE:** We changed the title as suggested.

Line 16: Replace to “This study aimed to evaluate the relationship among maternal residual feed intake (RFI) with growth performance and expression of genes involved in lipid metabolism in the offspring”.

**RESPONSE:** We changed the sentence as suggested.

Line 18: Replace to “Fifty-two cows previously classified as negative or positive RFI by genomic prediction were exposed to fixed-time artificial insemination (FTAI) protocols at 2 and 3 years of age using semen from the same bull”.

**RESPONSE:** We changed the sentence as suggested.

Line 21: Feedlot or drylot pens?

**RESPONSE:** Cows nursed the calves under feedlot conditions.

Line 22: “Cows were weighed every 28 days” for how long?

**RESPONSE:** The information was added as suggested.

Line 23 and 24: When?

**RESPONSE:** The information was added as suggested.

Line 23 to 25: Sentences could be improved.

**RESPONSE:** The sentences were corrected for better understanding.

Line 25: Why analyzed by experiment? It was never mentioned in the abstract there is more than one study.

**RESPONSE:** We replaced the word experiment by year. We mentioned above that two consecutive pregnancies of the cows were studied.

Line 26: Year needs to be in the model (if experiment was repeated within years. This information is not clear though).

**RESPONSE:** We corrected the sentence for better understanding.

Line 27: To know if there is an effect of year, it needs to be in the fixed model. According to line 26, year is not in the model. Please, address this discrepancy.

**RESPONSE:** We have used the word experiment as a synonym of year, which did not facilitate understanding. It was corrected to improve the readability. Thank you for warning!

Line 27: Growth, reproductive performance?

**RESPONSE:** The sentence has been improved for better understanding.

Line 31: It seems that the data was analyzed separately. Why?

**RESPONSE:** A previous approach of the data was carried out having both years analyzed as a single experiment, however the present approach was more efficient in showing the effect of the cow RFI class on the lipogenesis of calves, which is the main objective of the present study.

Line 42 to 43: Poor sentence. It can be more knowledgeable.

**RESPONSE:** The sentence was corrected as suggested.

Line 46 to 48: Needs to be rewritten. Because of the low nutrient availability?

**RESPONSE:** The sentence was rewritten as suggested.

Line 51: Replace to “can result in impaired growth rates…”

**RESPONSE:** It was replaced as suggested.

Line 56: and enhance sustainability of production systems.

**RESPONSE:** The sentence was corrected as suggested.

Line 67: Are authors referring to replacement heifers?

**RESPONSE:** In general, females (not just the heifers) are the animal category that remains longest in the herd. Having feed efficient females is a strategy to control the costs and enhance sustainability of production systems.

Introduction: This reviewer thinks that in the introduction more information on lipid metabolism needs to be added. For instance, why is your research focused on lipid metabolism?

**RESPONSE:** We inserted information about the importance of calves’ body composition, related to lipid metabolism, in introduction section as suggested.

Line 82: were conducted at the…

**RESPONSE:** The sentence was corrected as suggested.

Line 95 and 96: How above and below the average. Suggest adding the SD used.

**RESPONSE:** We separated the animals in two classes (negative and positive RFI). All animals above the mean were classified as positive RFI, and all animals below the mean were classified as negative RFI. RFI SD was presented in Table 5. We have rewritten the sentence for better understanding.

Line 102: Add the days of the experiment (d 0, d 28..). Were the AI different form exp 1 to exp 2? Details on them and add BW of cows and age in days. How were the cows weighed? Shrunk?

**RESPONSE:** No, AI was performed using the same strategy in both years. The difference is that in the second year, the cows were no longer primiparous but secundiparous. Body weight, age and weighing details were included as suggested.

Line 116: The calves were weight at birth and the birth weight was recorded. Suggest changing this sentence.

**RESPONSE:** The sentence was rewritten as suggested.

Line 118 to 120: The weight at 120 days of age (W120) was calculated from the birth weight and weight gain until days of age. Similarly, the weight at 210 days of age (W210) was calculated from the birth weight and weight gain until 210 days of age. The weight or ADG? Authors weighted cattle, so why was weight calculated?

**RESPONSE:** W120 and W210 were calculated based on birth weight and ADG calculated by regression based on every 28 d weighing of the calves. We weighted cattle but not exactly at 120 days or 210 days of age. These are standardized weights for specific ages.

Line 126: Were all the cows in the same pasture?

**RESPONSE:** Yes, all the cows were kept in the same pasture.

Line 133: Why were the forage samples divided and only the leaves analyzed? Cows also consume some roots, stem and so on. Hand samples should be collected in a way to represent what is consumed.
**RESPONSE:** The forage samples were divided into leaves, stems and dead material for better definition of the pasture situation in which the cows remained during pregnancy. Yes, we agree cows also consume other fractions, but unfortunately simulated grazing samples were not collected. We decided to present leaf composition, because it is the composition of the largest fraction of the material consumed by animals.

Line 149: This methodology does not seem okay because forage mass in one part of the pasture may not be the same in the other area. To estimate forage mass, it is more common to use regression equation using the double sampling technique. Forage availability was calculated as the amount of forage, in kg DM, available 150 within 1 m2 multiplied by 10,000 m2.

**RESPONSE:** Yes, it was done. We corrected the sentence for better understanding. Thank you.

Line 150: Why during the second trimester? Cows were grazing a pasture with less than 6% of CP. No supplementation was provided?

**RESPONSE:** Forage sampling was carried out to determine the chemical composition of the pasture in the months corresponding to the second gestational trimester of the cows, which is the critical period where nutritional restriction can have long term effects on growth potential of the calves. Mineral supplementation was provided to cows, as described in L 132-133.

Line 155: How many pens?

**RESPONSE:** All animals were kept in the same pen. The information was included as suggested.

Line 168 to 170: This sentence needs to be rewritten. It is not clear.

**RESPONSE:** As suggested, the sentence was rewritten for better understanding.

Line 176: Cow-calf pairs

**RESPONSE:** It was corrected as suggested.

Line 193: Where is this equation from? Citation needed.

**RESPONSE:** The citation [20] was included as suggested.

Line 279: Why the P-value is different for gene expression? Why is the AI in the fixed model? Where the protocols different both experiments? What were the random effects? Why if sex is the fixed term for calf variables why it is not interacting with each other (RFI|SEX)?

**RESPONSE:** Considering and discussing trends with P < 0.15 in gene expression analyzes is well accepted in the literature, due to the subtlety of the variables. AI was included as fixed effect in the model for adjustment because we had two AI protocols in each year, which implies a difference of approximately 40 days of age between calves from the first and second protocols. Maternal RFI x calf sex interaction was tested, and because it was not significant, was excluded from the model. This information is present in the text L 279-280.

Line 297: Deducted instead of deduced? Why to have a descriptive table?

**RESPONSE:** It was corrected as suggested. We decided to keep the descriptive table, as its information helps in the results interpretation and in the data variability evaluation.

Table 6: Needs more information. For example, how long was this DMI measured in calves (pre-weaning or post-weaning). Those details need to be in the table.

**RESPONSE:** DMI was measured in calves pre-weaning (from 35 days of age until weaning). This is described in Material and Methods section L 174-176.

**Reviewer: 2**

**Comments to the Author**

Overview: This study evaluated cow-calf pairs until weaning in two experiments. Cows with different RFI and different production systems were evaluated. Animal performance and gene expression was used to assessed.

Major comments: My major consideration in this study is the potential carry-over effect of the higher weight of the cows in the second experiment compared to the first. Although cows were managed with the calves under grazing conditions in the second experiment, body weight could partly compensate nutrition for gestation. This needs to be addressed in the ‘discussion’ section. Please, provide how ADG was calculated for each animal category. Please, improve the resolution of figures.

**RESPONSE:** We appreciated all the efforts you made to improve the quality of this manuscript. Thank you. As suggested, we included the consideration about body weight could partly compensate nutrition for gestation in the Discussion. We also provided the description of how ADG was calculated in Material and Methods section.

L056: the citation is incorrect;

**RESPONSE:** We corrected the citation.

L072: the citation is incorrect;

**RESPONSE:** We corrected the citation.

L075-079: in my opinion, the hypothesis and the objectives are somewhat disconnected, especially with the sentence ‘genes related to lipid metabolism’;

**RESPONSE:** We adjusted the hypothesis to be more connected to the objective.

L082: ‘conducted at Centro’ instead of ‘conducted Centro’;

**RESPONSE:** The sentence was corrected as suggested.

L093: provide the country of the company. Please correct this throughout the text;

**RESPONSE:** The information was included as suggested.

L107: muscle nomenclature is incorrect;

**RESPONSE:** We corrected the muscle nomenclature as suggested.

L108: please provide information of the manufacturer;

**RESPONSE:** The information was included as suggested.

L116: ‘The calves were weighed’ instead of ‘The calves were weight’;

**RESPONSE:** The sentence was corrected as suggested.

L120-121: provide a mean number with standard deviation for calves’ weaning;

**RESPONSE:** The information was included as suggested.

L129-130: provide information on how the area was sampled, e.g. which height off the ground, etc.;

**RESPONSE:** The information was included as suggested.

L141: the citation is incorrect;

**RESPONSE:** We corrected the citation.

L143: the citation is incorrect;

**RESPONSE:** We corrected the citation.

L143: the citation is incorrect;

**RESPONSE:** We corrected the citation.

L144: the citation is incorrect;

**RESPONSE:** We corrected the citation.

L147: the citation is incorrect;

**RESPONSE:** We corrected the citation.

L158: provide the country of the company;

**RESPONSE:** The information was included as suggested.

L201: muscle nomenclature is incorrect;

**RESPONSE:** We corrected the muscle nomenclature as suggested.

L208: ‘obtain’ instead of ‘obtained’;

**RESPONSE:** The sentence was corrected as suggested.

L209: ‘sutured’ instead of ‘closed’;

**RESPONSE:** The sentence was corrected as suggested.

L210: ‘few days’ is not scientific. Please, be assertive;

**RESPONSE:** We corrected the sentence to be more assertive as suggested.

L216: muscle nomenclature is incorrect;

**RESPONSE:** We corrected the muscle nomenclature as suggested.

L236: ‘totaling’ instead of ‘total’;

**RESPONSE:** The sentence was corrected as suggested.

L237: the citation is incorrect;

**RESPONSE:** The citation was corrected.

L263: the citation is incorrect;

**RESPONSE:** The citation was corrected.

L264: the citation is incorrect;

**RESPONSE:** The citation was corrected.

L355: muscle nomenclature is incorrect;

**RESPONSE:** We corrected the muscle nomenclature as suggested.

L355: ‘from’ instead of ‘to’;

**RESPONSE:** We decided to keep the expression “born to” in the whole text.

L374: I do not understand what authors mean with this;

**RESPONSE:** We corrected the sentence for better understanding.

L382: muscle nomenclature is incorrect;

**RESPONSE:** We corrected the muscle nomenclature as suggested.

L383: ‘from’ instead of ‘to’

**RESPONSE:** We decided to keep the expression “born to” in the whole text.

L383: avoid terms such as ‘very important’;

**RESPONSE:** We corrected the sentence excluding the term as suggested.

L393: include a reference to support the affirmation;

**RESPONSE:** The reference [27] was included as suggested.

L394: the citation is incorrect;

**RESPONSE:** The citation was corrected.

L401: the citation is incorrect;

**RESPONSE:** The citation was corrected.

L401-403: rewrite this sentence;

**RESPONSE:** The sentence was rewritten for better understanding.

L403: why were you RFI variation lower compared to these other studies? Breed, animals’ weight, and diet were similar?

**RESPONSE:** The cows of the present study are extremely homogeneous, because they came from a herd that has been selected for growth since 1980 and for RFI since 2010. For this reason the variability detected in phenotypic RFI is lower compared to result found in the literature.

L403: the citation is incorrect;

**RESPONSE:** The citation was corrected.

L413: the citation is incorrect;

**RESPONSE:** The citation was corrected.

L418-420: what about the physiological differences between cows?;

**RESPONSE:** We included this point of view as suggested.

L426: the citation is incorrect;

**RESPONSE:** The citation was corrected.

L426-427: what were the conditions of the experiment compared to this study?;

**RESPONSE:**  The conditions comparing the both years of this study were opposite.

L439: the citation is incorrect;

**RESPONSE:** The citation was corrected.

L443: the citation is incorrect;

**RESPONSE:** The citation was corrected.

L444: ‘from’ instead of ‘to’;

**RESPONSE:** We decided to keep the expression “born to” in the whole text.

L450: the citation is incorrect;

**RESPONSE:** The citation was corrected.

L455: the citation is incorrect;

**RESPONSE:** The citation was corrected.

L466: the citation is incorrect;

**RESPONSE:** The citation was corrected.

L473: avoid terms such as ‘extremely important’;

**RESPONSE:** The sentence was corrected as suggested.

L512: ‘global’ instead of ‘national’;

**RESPONSE:** The sentence was corrected as suggested.

L554: avoid using references which are not in English;

**RESPONSE:** Some of our references were not available in English.

L564: check the references. For example, the journal in the citation is not abbreviated.

**RESPONSE:** All the references were checked. The names of the journals were corrected. Thank you.
