# Supplementary material for: Performance and muscle lipogenesis of calves born to Nellore cows with different residual feed intake classification
Source: PLoS One. 2022 Jul 29;17(7):e0272236. doi: 10.1371/journal.pone.0272236 (PMC9337683; doi:10.1371/journal.pone.0272236)
Supplement: Attachment — Submitted filename: Paz et al 2022_responses to reviewers.docx [file pone.0272236.s004.docx]

**RESPONSES TO REVIEWERS**

Manuscript ID PONE-D-21-37544R1:

"Performance and muscle lipogenesis of calves born to Nellore cows with different residual feed intake classification"

Dear Dr. Marcio Duarte,

We appreciated all the efforts you and the reviewers made to improve the quality of this manuscript. Below are the responses for each comment and suggestion made. The changes in the text are highlighted using Word track changes tool in marked-up copy.

Thanks a lot,

The authors

**Reviewer(s)' Comments to Author:**

**Reviewer #2:**

Major comments:

The manuscript has improved from its early version. However, several issues are pending, some more were raised, and some comments were not addressed at all. For example, the resolution of figures is still the same.

**RESPONSE:** We appreciated all the efforts you made to improve the quality of this manuscript. And we apologize for the frustrations while checking our correction for the last revision. We hope we were more assertive this time. About the resolution of the figures, we are complying with the tiff format required by the journal, and when the submission system transforms tiff into pdf, the resolution fails. We are in contact with the editorial board to solve this problem. Thank you.

Minor comments:

L019: fifty-two, or fifty-three? Check L094;

**RESPONSE:** There was a mistake here. It is correct now. Thank you.

L020: it is important to state that two FTAI protocol were used;

**RESPONSE:** Yes, we agree. But the space is limited in abstract section. This information is fully descripted in Material and Methods section.

L024-25: check this sentence;

**RESPONSE:** We checked and modified the sentence as suggested.

L059: there are several entries for ‘body weight’, thus I suggest abbreviating it;

**RESPONSE:** It was done as suggested.

L072: although it was changed, the phrase ‘Results showed that’ could be improved;

**RESPONSE:** We corrected the sentence as suggested.

L101-106: the manuscript would benefit if authors could clarify as to why three extra cows were added in the second year of the experiment, and also if the 25 cows were all used in the second year;

**RESPONSE:** We had a contemporary group of cows that were exposed to the same conditions in breeding seasons for 2 consecutive years. Those who became pregnant entered the study. Some became pregnant only in the first year, others only in the second year, and most in both years. The sentence was modified for better understanding.

L129: ‘(i.e. LEA, BF, and RF)’ instead of ‘(LEA, BF, and RF)’;

**RESPONSE:** It was corrected as suggested.

L133: ‘During pregnancy of year 1’ instead of ‘During pregnancy’;

**RESPONSE:** It was corrected as suggested.

L133-134: include the pasture management regimen, area and other relevant information, such as fertilization. Also, I suggest clarifying if all cows were kept in the same paddock;

**RESPONSE:** The information was included as suggested.

L145: my previous request as completely overlooked, which is frustrating. Provide the country of the company. Please correct this throughout the text;

**RESPONSE:** We apologize for the mistake. We provided the name of the company, city, state and country for all equipment.

L145-154: the citations in this paragraph are mostly incorrect. Authors have modified them in the current version, but they are still incorrect;

**RESPONSE:** We corrected the citations again. We did not understand exactly what was incorrect. Was the official method for specific analyzes? Is it preferable not to cite the method, just the reference?

L146: the numeral should have one space of the degree symbol;

**RESPONSE:** We added the space as suggested.

L150: ‘ash’ instead of ‘ashes’;

**RESPONSE:** We corrected as suggested.

L155: the term ‘in kg DM’ is incorrect;

**RESPONSE:** We corrected the sentence as suggested.

L157: ‘t DM/ha’ is incorrect;

**RESPONSE:** We corrected the sentence as suggested.

L165: provide the full list of information for the system;

**RESPONSE:** It was provided as suggested.

L168: how was feed offering adjusted daily if leftovers were not evaluated daily?;

**RESPONSE:** The amount of feed offered was adjusted daily to keep leftovers around 10% (to offer more or less feed). There must be daily leftovers to ensure that feed intake has been ad libitum, but excessive leftovers damage experimental management.

L171: ‘for the leaf fraction’ instead of ‘in the previous item’;

**RESPONSE:** It was corrected as suggested.

L184: be specific and modify ‘in the previous item’;

**RESPONSE:** It was modified as suggested.

L187-191: include the pasture management regimen, area and other relevant information, such as fertilization;

**RESPONSE:** It was included as suggested.

L196: ‘t DM/ha’ is incorrect;

**RESPONSE:** We corrected the sentence as suggested.

L208: indicate right side of what;

**RESPONSE:** It was included as suggested.

L251: if the work is not published, do not use it as literature, as it was not peer reviewed;

**RESPONSE:** We excluded the thesis citation as suggested.

L260: the numeral should have one space of the degree symbol;

**RESPONSE:** The space was added as suggested.

L267: if the work is not published, do not use it as literature, as it was not peer reviewed. Furthermore, the text is in a language other than English, which does not fit as a reference for the methodological part of the study;

**RESPONSE:** We excluded the thesis citation as suggested.

L345-347: the difference was only for the FABP4 gene, and the others had a tendency. The sentence should be rewritten;

**RESPONSE:** The sentence was rewritten as suggested.

L402-403: the referencing in this sentence is odd;

**RESPONSE:** The sentence was modified for better understanding.

L415: ‘interferes’ instead of ‘interfere’;

**RESPONSE:** It was corrected as suggested.

Figure 1: ‘Percent’ instead of ‘Porcent’;

**RESPONSE:** It was corrected as suggested.

Figure 1: ‘SCD1’ instead of ‘SCD’;

**RESPONSE:** It was corrected as suggested.

Figure 2: ‘SCD1’ instead of ‘SCD’.

**RESPONSE:** It was corrected as suggested.

Reviewer #3: This manuscript presents the results from two studies/years conducted to evaluate the effect of maternal RFI on calves’ performance and muscle gene expression. One of the limitations of this study is that the groups of cows were very similar in terms of RFI and the authors observed only a trend for this trait as well as the variables evaluated on claves. I am not convinced that the data was analyzed in the best way. Authors should describe better why FTAI was included in the model. The manuscript language is understandable, but it could be improved.

**RESPONSE:** We appreciated all the efforts you made to improve the quality of this manuscript. Thank you. The study was performed with a contemporary group of a homogeneous Nellore herd, selected for growth and RFI. This is why the cows were very similar in terms of RFI, which brings us some advantages and other disadvantages. A previous approach of the data was carried out having both years analyzed as a single experiment, and as repeated measures over time, however the present approach was more efficient in showing the effect of the cow RFI class on the lipogenesis of calves, which is the main objective of the present study. Overall, we improved writing style and included the missing information as suggested.

Overall questions:

1. Was the same group of cows used in both years? Should we consider these as two different experiments or the same experiment repeated for two consecutive years?

**RESPONSE:** Yes, the same group of contemporary cows were used in both years. Considering the data as two different experiments was more efficient in showing the effect of the cow RFI class on the lipogenesis of calves, which is the main objective of the present study.

2. How can the authors justify the lack of differences between groups for RFI?

**RESPONSE:** The study was performed with a contemporary group of a homogeneous Nellore herd, selected for growth and RFI.

Specific comments:

L. 25 – carcass?

**RESPONSE:** We corrected the sentence as suggested.

L. 25-26 . “Muscle gene expression was evaluated in calves at birth and weaning”

**RESPONSE:** We corrected the sentence as suggested.

L. 27- Why FTAI is a factor? Was it different?

**RESPONSE:** FTAI was included as fixed effect in the model because we had two AI protocols in each year (in the same breeding season), which implies a difference of approximately 40 days of age between calves from the first and second protocols. This is not detailed in abstract section, because of the space limitation (max 300 words), but the information is in material and methods section.

L. 28-29 – “There was no effect of maternal RFI on calves ADG in the first year.”

**RESPONSE:** We corrected the sentence as suggested.

L. 36-38 – This conclusion sentence could be more specific to the current study

**RESPONSE:** We modified the sentence to be more specific.

Introduction – The sentences could be more connected making the text more pleasant to read. I think the introduction could bring some information regarding the heritability of RFI and other characteristics evaluated in this study.

**RESPONSE:** We tried to improve the readability of the introduction section, modifying the text.

L. 49-50. “received and recorded by the genome” I think this sentence is strange. Please review it.

**RESPONSE:** We modified the sentence as suggested.

L.54-56 – This is a long and wordy sentence.

**RESPONSE:** We modified the sentence as suggested.

L. 61-65 . Again this is a long sentence and hard to follow. Please review it.

**RESPONSE:** The sentence was modified as suggested.

L.96-97. How good is this genomic prediction? Was the RFI evaluated or only predicted?

**RESPONSE:** The genomic prediction of RFI is reliable and has been widely used due to the difficulty and cost of phenotypic measurement of the trait. We have the phenotypic RFI of these cows, but it was measured in adult animals and not post-weaning as recommended. For this reason we opted for the genomic prediction.

L. 109 – Ribeye area (REA) and backfat thickness (BFT) is more commonly used for cattle.

**RESPONSE:** We corrected as suggested.

L. 113-114. Please, provide more details regarding the equipment and probe used as well as the technician's ability and/or certification to collect and analyze the images.

**RESPONSE:** The information was included as suggested.

L. 135. Why not all gestation or the last two trimesters of gestation?

**RESPONSE:** Because it is in the second trimester of the gestation that muscle and lipid tissue cells are formed. For future studies, we will evaluate the entire pregnancy, stratifying into 3 trimesters.

L. 171- Please reword this sentence removing “previous item”

**RESPONSE:** The sentence was modified as suggested.

L. 184. Please, be more specific here. What do you mean by “previous item”

**RESPONSE:** The sentence was corrected as suggested.

Table 1 and 3. Would be possible to concatenate table 1 and 3? DM and TDN are as a % of what?

**RESPONSE:** We believe that keeping tables 1 and 3 separate makes it easier to understand, since we are talking about very different phases of the study (first pregnancy - Table 1; lactation of the second calf -Table 2).

L. 213- Please delete “An”

**RESPONSE:** It was deleted as suggested.

L. 217. I suggest deleting “was complete”

**RESPONSE:** It was deleted as suggested.

L. 220-224. Where were these muscle samples removed from?

**RESPONSE:** We included the information as suggested.

L. 228 – What was done with these samples?

**RESPONSE:** The samples were taken for histological analysis. The methodology is described in L 262-273. We modified the text for better understanding.

L. 275- Again, why FTAI is a factor? Was it different?

**RESPONSE:** FTAI was included as fixed effect in the model because we had two AI protocols in each year (in the same breeding season), which implies a difference of approximately 40 days of age between calves from the first and second protocols. It is described in L 105-110.

L. 275. In my opinion, calves and cow BW should be evaluated as repeated measures.

**RESPONSE:** A previous approach of the data was carried out having performance of cows and calves analyzed as repeated measures. But considering the data as two different experiments was more efficient in showing the effect of the cow RFI class on the lipogenesis of calves, which is the main objective of the present study.

L. 278. I couldn’t understand it. All data was not normally distributed?

**RESPONSE:** This is a protocol of evaluation of gene expression data.

L. 300. Did you evaluate fetal weight?

**RESPONSE:** No, unfortunately we didn´t.

L. 332- “efficient”

**RESPONSE:** We corrected as suggested.

Figures. Please review the axis label “porcent”

**RESPONSE:** It was corrected as suggested.

Table 7. Where is the DMI data for cows?

**RESPONSE:** The cows nursed their second calves under pasture conditions. For this reason we do not have DMI data in table 7.

L. 400-402. Where is this data? Table 6 is showing a 0.1kg difference for year 1.

**RESPONSE:** Data from Year 1 is in table 6 and from Year 2 is in table 7.

L. 400-407. So, how about the current study? Why the difference was so small and only tended to differ?

**RESPONSE:** We modified the sentence, completing the information.

L. 422 – I think “weaning efficiency” would be a better term than “productive efficiency”. Please consider replacing it across the manuscript.

**RESPONSE:** We replaced the term as suggested.

L. 428-433. This sentence is too long and confusing. Please rephrase it.

**RESPONSE:** We rewritten the phrase as suggested.

L. 444 – These are not body composition data. Please review it throughout the manuscript.

**RESPONSE:** It was corrected as suggested.

L.471. delete enzyme or protein. It is redundant

**RESPONSE:** It was deleted as suggested.

L. 488-489. I am not sure if this sentence is accurate. In my understanding, adipogenesis occurs throughout the animal’s life. Please review this sentence.

**RESPONSE:** The sentence was rewritten as suggested.
